# A Method of Deep Learning Model Optimization for Image Classification on Edge Device

**DOI:** 10.3390/s22197344

**Published:** 2022-09-27

**Authors:** Hyungkeuk Lee, NamKyung Lee, Sungjin Lee

**Affiliations:** 1 Media Intelligence Research Section, Electronics and Telecommunications Research Institute, 218, Gajeong-ro, Yuseong-gu, Daejeon 34129, Korea; 2 Electronic Engineering, Dong Seoul University, 76 Bokjeong-ro, Sujeong-gu, Seongnam-si 13117, Korea

**Keywords:** image classification, lightweight network, network compression, convolutional neural network, quantization, pruning, knowledge distillation

## Abstract

Due to the recent increasing utilization of deep learning models on edge devices, the industry demand for Deep Learning Model Optimization (DLMO) is also increasing. This paper derives a usage strategy of DLMO based on the performance evaluation through light convolution, quantization, pruning techniques and knowledge distillation, known to be excellent in reducing memory size and operation delay with a minimal accuracy drop. Through experiments regarding image classification, we derive possible and optimal strategies to apply deep learning into Internet of Things (IoT) or tiny embedded devices. In particular, strategies for DLMO technology most suitable for each on-device Artificial Intelligence (AI) service are proposed in terms of performance factors. In this paper, we suggest a possible solution of the most rational algorithm under very limited resource environments by utilizing mature deep learning methodologies.

## 1. Introduction

As athe demand for applying deep learning models on mobile and Internet-of-Things (IoT) devices increases, industrial needs for a Deep Learning Model Optimization (DLMO) and Neural Network Compression (NCC) suitable for on-device Artificial Intelligence (AI) are also increasing. In particular, an AI service on the edge devices, referred to as edge computing or Artificial Internet of Things (AIoT) [1], is being applied in various fields such as a smart cities, smart factories, smart agriculture, smart mobility, etc. Since the native neural networks are difficult to deploy on tiny devices and embedded systems with limited resources, researchers in this field have studied model optimization and network compression [2]. Many studies have tried to apply deep learning into several applications such as the detection of diabetic retinopathy [3], the management of security in Internet of Medical Things (IoMT) environments [4], and optimization techniques for IoT data [5,6]. Due to model optimization and network compression, the memory size and a computational delay of deep learning models are reduced compared to the native neural networks, while the performance of models is maintained as well [7].

In this paper, we address usage strategies of DLMO based on performance evaluation through several combinations of Lightweight Convolution, Quantization and Pruning techniques. First, in order to examine each performance among combinations, we are using VGGNet [8] and ResNet [9] as baseline networks. Then, for a comparison, MobileNet v1, v2 and v3 [10,11,12] are used as lightweight networks. In various IoT use cases, we evaluate the performances of several quantization techniques, which comprise Quantization Aware Training (QAT) and subtypes of Post Training Quantization (PTQ), i.e., Baseline Quantization (BLQ), Full Integer Quantization (FIQ) and Float 16 Quantization (F16) [13,14]. Lastly, for the pruning technique, the performance improvement will be analyzed by applying the training method to the basic Convolution Neural Network (CNN) and lightweight CNN technologies [14,15]. As datasets for performance analysis, we will use the Canadian Institute For Advanced Research 10 (CIFAR10) and CIFAR100. Rather than present a new high-level algorithm, we try to guide a possible combination of the most rational algorithm under very limited resource environments by utilizing the dominant technologies with high maturity.

The remainder of this paper is organized as follows. Section 2 presents the related work. Section 3 illustrates the proposed methodology with respect to lightweight network techniques, while Section 4 shows the simulation results. Section 5 provides the conclusions of the work.

## 2. Related Work

The performance improvement in the deep learning-based image classification models has come from the excellence of the CNN’s feature extraction [16]. Thus, how to design the network layer as an extractor’s role has become key to improving the overall performance and computational efficiency. In the early days of deep learning, the convolutional extractor network was mainly focused on performance improvement, so the number of layers gradually increased and the structure was designed to be more complex. Nevertheless, the authors in [17,18] pointed out that a bottleneck in CNN’s performance is due to the imbalanced memory distribution in CNN designs, i.e., the first several blocks have an order-of-magnitude larger memory usage than the rest of the network. However, even though VGGNet [8], with a simple 3×3 convolutional block-based structure, has dramatically reduced computational complexity, it showed a comparable performance to the Inception model [19] with a complex structure. Then, an interest in reducing computational efficiency of convolution extractor networks has begun to rise. Afterwards, ResNet, with a skip-connection structure [9], also contributed to both a reduction in computational complexity and an improvement in accuracy. In addition, enhanced versions of ResNet appeared, such as Wide Residual Network (WRN) [20] and ResNeXt [21]. In this trend, as subtypes of MobileNets (MobileNet v1 using Depthwise Separable Convolution [10], MobileNet v2 [11] using Bottleneck Residual Block [9] and Squeeze and Excitation Block [22], MobileNet v3 [12] using Network Architecture Search (NAS) [23]) appear, convolution lightweight technology has made substantial progress.

The authors in [24] systematically studied model scaling and identified that carefully balancing network depth, width and resolution can lead to better performance. They proposed a new scaling method that uniformly scales all dimensions of depth/width/resolution using a simple yet highly effective compound coefficient. The authors in [25] improved the performance by combining ResNet scaling strategies. The strategy depends on the training regime and offers two new scaling strategies: (1) scale model depth in regimes where overfitting can occur (width scaling is preferable otherwise); (2) increase image resolution more slowly. The Google brain team has thoroughly researched a method to reduce significant amounts of computational resources, memory and power to train and run on mobile and IoT devices. Google provided one of the core Machine Learning (ML) kits, ‘Learn2Compress [26]’, to make machine learning accessible for all mobile developers. It is an automatic model compression service and enables custom on-device deep learning models in TensorFlow Lite that run efficiently on mobile devices, without developers having to worry about optimizing for memory and speed. TensorFlow Lite Micro (TFLM) is, in particular, an open-source ML inference framework for the tiny embedded systems [27]. Other AI industries have also provided network compression and light weight ML models such as the Pytorch module, NeuralMagic, Nvidia’s TensorRT, OpenVINO, etc. The authors in [28] demonstrated the efficient neural network kernel to maximize the performance and minimize the memory consumption on Arm Cortex-M processor. On microcontrollers, which are small computing resources on a single VLSI integrated circuit (IC) chip for IoT or embedded devices, the authors in [17,18] suggested a joint framework of the efficient neural architecture and the lightweight inference engine for image classification. There have been also vigorous studies on reducing the amount of memory consumption or managing memory resources efficiently in [29,30,31]. We have summarized related works mentioned above in Table 1.

## 3. System Model

### 3.1. Quantization Technique

In [2], a model quantization was a widely used technique to compress and accelerate the inference stage of deep learning. Recent hardware accelerators for deep learning have begun to support mixed precision (1–8 bits) to further improve computation efficiency, which raises a great challenge to find the optimal bitwidth for each layer: it requires domain experts to explore the vast design space, trading off between accuracy, latency, energy, and model size, which is both time-consuming and sub-optimal.

A quantization technique compresses the network weights by reducing the number of bits, and then the network weight becomes smaller from 32 bits. Therefore, the quantization method limits the dynamic range and the expression accuracy of bits but also has the advantage of reducing the overall network weight size as much as the number of quantized bits.

To analyze the quantization transformation mathematically, let us define the former FP32-bit tensor as $x_{f}$ and the quantized INT8 tensor as $x_{q}$. The basic transformation method of quantization then becomes:

(1) $$\begin{array}{c} x_{q}=Round\left(s\cdot Clip\left(x_{f},-r,r\right)\right),\;\mathrm{where}\;s=\frac{127}{r}. \end{array}$$

**Figure 1:**
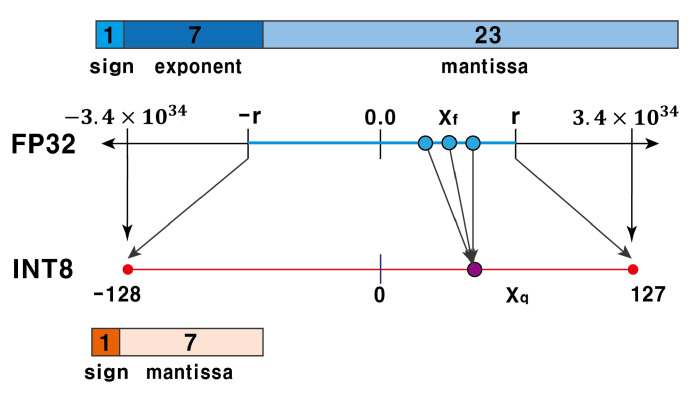
Quantization from FP32 to INT8.

As shown in Figure 1, the distribution of the dynamic range of FP32 is in $\left[-3.4\times 10^{-38},3.4\times 10^{38}\right]$ based on IEEE 754 standard [32] and the dynamic range of INT8 can express 255 equally spaced numbers. In other words, in order to map numbers from FP32 to INT8, the Clip function is used to discard some outlier numbers outside −r and *r* in the dynamic range of FP32. In addition, *s* is used for spacing adjustment. Therefore, the conversion from FP32 to INT8 results in some latency due to the operations of Equation (1), such as Clip, Round and Scale.

**Figure 2:**
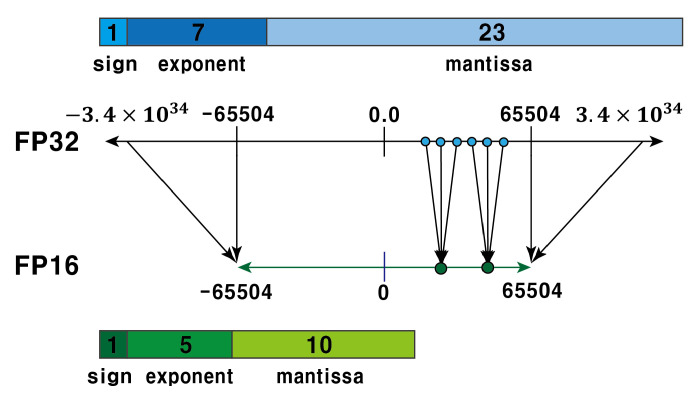
Quantization from FP32 to FP16.

On the other hand, the dynamic range of FP16 becomes [−65504,65504] with the half size of FP32 based on IEEE 754 standard [32]. As shown in Figure 2, the two floating point representations FP32 and FP16 shows the similarity in the format, so that their conversion needs just bitwise operation with little latency, i.e., the exponent and mantissa of FP16 can be obtained by removing 2 LSBs (Least Significant Bits) of the exponent in FP32 and 13 LSBs of mantissa in FP32.

The quantization technique can be divided into two types depending on whether the bit conversion is performed before or after training the weight values:

- *Post-Training Quantization(PTQ)*: A method of training weights with FP32 and then quantizing the results into smaller datatypes;
- *Quantization Aware Training (QAT)*: A method of training weights for maximizing their accuracy with the quantized datatype.

PTQ techniques can be also categorized into the following three methods according to how the weights are quantized: Baseline Quantization (BLQ), Full Integer Quantization (FIQ) and FP16 Quantization.

**Figure 3:**
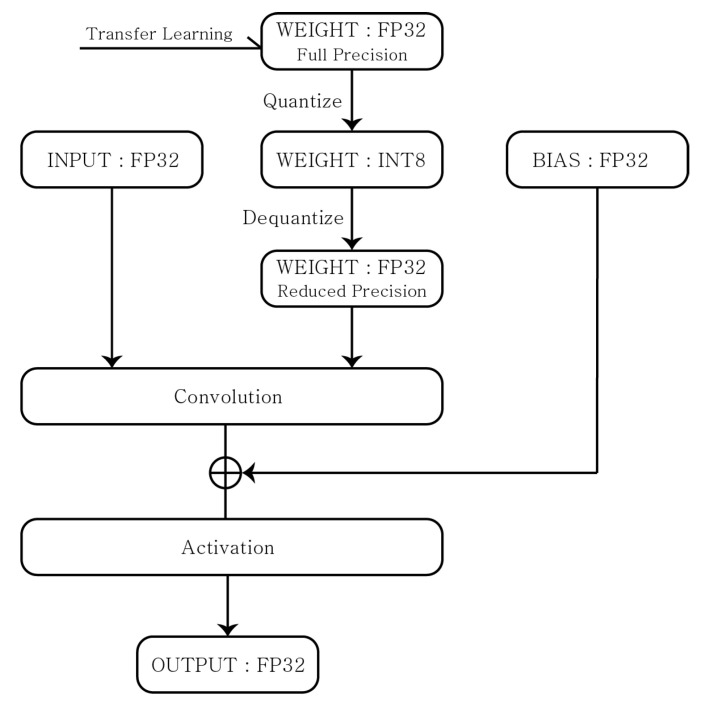
Baseline Quantization.

The BLQ is a method of quantizing FP32 weight values into the INT8 type. In particular, the inference is performed by reversing the quantized INT8 value to a FP32 decimal value with the reduced precision. Then, as shown in Figure 3, the inference is operated as:

(2) $$\begin{array}{c} Input(\mathrm{FP}32)\times Weight(\mathrm{FP}32\;\mathrm{with}\;\mathrm{reduced}\;\mathrm{precision})\\ +Bias(\mathrm{FP}32)=Output(FP32). \end{array}$$

**Figure 4:**
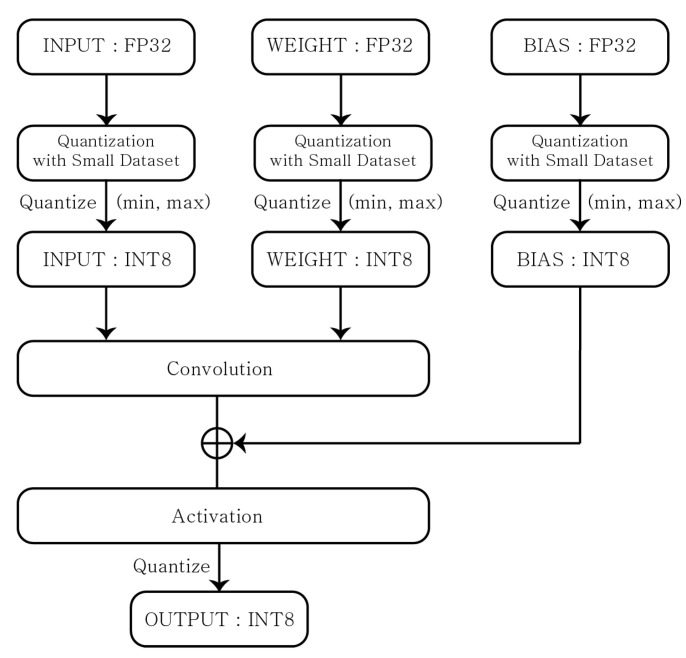
Full Integer Quantization.

The FIQ is a method that quantizes all mathematical values of the network model using a sample data set, that is, it determines the quantization parameters such as the minimum and maximum values of the weight and activation function values and the bias values. Then, the inference is performed by quantizing input FP32 values to INT8 values with the predetermined quantization parameters. Here, to determine the quantization parameters, a small data set of around 100–500 samples is needed for the additional training. Figure 4 represents the procedure.

**Figure 5:**
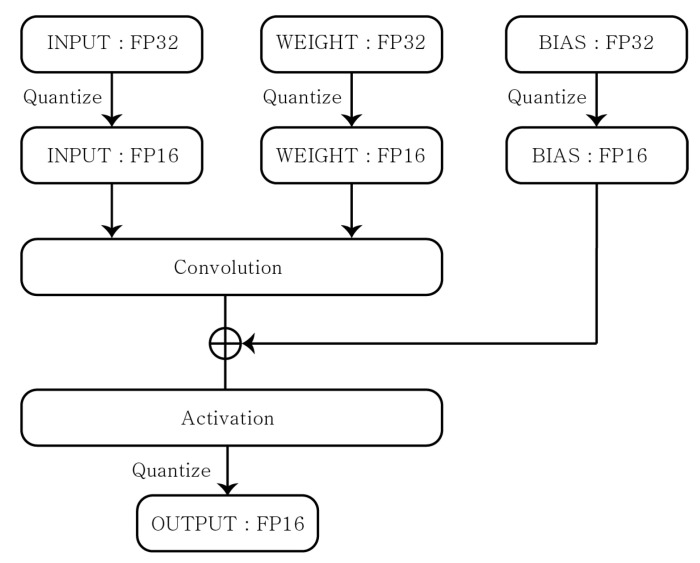
Float 16 Quantization.

The FIQ also includes an implementation of the float fallback method in case there is no implementation of conversion to integer values for each decimal value due to hardware limitations. The F16 is a method of expressing the FP32 weight values into to the nearest FP16 weight values with lower precision. It is possible to reduce the model size in half with minimal accuracy loss. The overall procedure is shown in Figure 5.

### 3.2. Pruning

The pruning technique is a method to leave weights over the threshold value and set the rest of weights to zero within a deep learning operation. In general, a rule for choosing pruning weights is to sort some weights by their absolute values and to adjust the rest of weights to zero the smallest weights until some desired sparsity level is satisfied [15]. In this paper, weights set to zero in the aforementioned CNN models are gradually increased for 60–70 iterations to achieve maximal accuracy.

### 3.3. Knowledge Distillation

Knowledge Distillation (KD) is a deep learning method to transfer the knowledge from the cumbersome model with the large deep neural network to a small model with more suitable structure for deployment [33]. The cumbersome model and the small model are also known as teacher model and student model, respectively. A simple way to transfer the generalization ability of the teacher model to the student model is to use the class probabilities produced by the teacher model as “soft targets” for training the student model.

In KD, the soft target, i.e., each class probability $q_{i}$ from each logit $z_{i}$ in a “softmax” equation, can be more distributed through temperature *T* as:

(3) $$\begin{array}{c} q_{i}=\frac{exp(z_{i}/T)}{\sum_{j}exp\left(z_{j}/T\right)} \end{array}$$

Since the vanilla KD method was published in [33], various KD schemes have been developed. As shown in Table 2, Table 3 and Table 4, the KD methods can be generally classified in terms of Knowledge Type (KT), Distillation Type (DT) and Teacher–Student Architecture (TSA) [34]. First of all, the KT for KD can be classified into response, feature and relation according to the KT, as shown in Table 2. The aim of the response-based KT is to calculate the distillation loss from the logit outputs of the teacher and student models [35,36,37,38]. The feature-based knowledge type aims to calculate the distillation loss from the intermediate representations of the teacher and student models [39,40,41,42,43,44]. The relation-based knowledge type aims to utilize the relation from the feature maps [45,46,47,48]. Although it has a similarity with the previous feature-based KT in the perspective of using the intermediate feature map, it is distinguished from using the manipulated function of the feature maps such as the Gram matrix [45].

On the other hand, the KD can be classified into online, offline and self-distillation according to the DT, as shown in Table 3. The offline-based DT aims to transfer the knowledge from a pre-trained teacher model into a student model [33,42,43,44,49,50,51]. The online-based DT aims to update the teacher and student models simultaneously [41,47,52,53,54,55]. In the special case of online distillation, the self-distillation-based distillation type aims to utilize the same network model for the teacher and student models [56,57,58,59,60,61].

Last, as shown in Table 4, the KD can be classified into the same architecture as the teacher [62,63,64]; reduced architecture from teacher and light-weight architecture using light-weight convolution modules [51,65,66]; and Quantization and Pruning according to the TSA [10,67,68,69,70,71,72]. In particular, the light-weight architecture-based student model is based on the various light-weight convolution structure mentioned in Section 2. Likewise, the quantization and pruning based student model is based on the structure mentioned in Section 3.1 and Section 3.2.

This paper evaluates the performance of the Vanilla KD with a Light-Network-based student architecture in terms of Response, Offline and Light Network types (MobileNetv1, v2 and v3).

## 4. Simulation Results

### 4.1. Performance of Quantization and Pruning

In order to examine the performance of the quantization and pruning technology groups, let us define the evaluation setups. First, the basic technology without quantization and pruning is denoted as NQ (No Quantization). Then, as mentioned in Section 3.1, the other quantization techniques for the evaluation are denoted by BLQ, FIQ, F16 Quantization and QAT.

In addition, let us define the pruning technique as PRN (Pruning). Then, a technique that applies both quantization BLQ and pruning is PRQ (Pruning Quantization). Finally, VGGNet and ResNet50, as the baseline networks, are tested on the CIFAR10 and CIFAR100 datasets. The recognition delay or latency (Lat) is the value computed for 10,000 images of the validation set in CIFAR10 and CIFAR100. All of these experiments have gone through the TensorFlow-Lite conversion and code optimization procedures [14].

As shown in the results of Table 5, Table 6 and Table 7, when the quantizations are applied, all quantization setups have similar or better accuracy performance (as shown in “Acc”) than NQ, whereas the model size (Size) is decreased by 25%∼50%. Among them, it is observed that BLQ, FIQ and QAT have similar performance, i.e., accuracy around 80% and size of 25%. However, F16 has a size of 50% with a similar accuracy around 80%. In terms of “Lat”, F16 shows the best performance. On the other hand, BLQ, FIQ and QAT have rather long latencies. This proves that the conversion from the FP32 inputs to INT8 values takes a significant amount of time.

For the performance of “PRN”, it is observed that although its accuracy and size are similar with those of the quantization techniques, it has significant advantages in “Lat”. The reason for this is that pruning has no conversion procedure from the FP32 input to other data units such as FP16 or INT8. Moreover, the density of effective weights, i.e., non-zero values, is sparse compared to that of the quantization, so the computational complexity can be reduced. Those two reasons are the main factors in the model compression performance of PRN.

In addition, if the quantization is added to this pruning technique, i.e., PRQ, the model compression performance can be extremely enhanced, but latency increases similar with the other quantization schemes due to the conversion of data units.

In particular, it is remarkable that pruning shows better accuracy in ResNet compared to VGGNet. From this fact, it can be seen that the higher the network depth and number of channels, the greater the pruning gain.

Based on the above experiments and observations, the following remarks can be derived:

**Remark** **1.** *If the quantization is applied, the model can be compressed while maintaining the similar accuracy.*

**Remark** **2.** *The data conversion procedure of the quantization can cause recognition delays.*

**Remark** **3.** *If pruning is applied, both the model size and the recognition delay can be reduced while maintaining a similar accuracy.*

**Remark** **4.** *The pruning shows a better performance improvement when applied to neural networks with a large capacity.*

### 4.2. Performance Evaluation of Light-Weight CNNs

In this section, we examine the performance of the lightweight CNNs, i.e., MobileNet v1, v2 and v3. As shown in Table 8, Table 9, Table 10 and Table 11, it is observed that MobileNet v2 shows higher accuracy at only 5∼10% the size of ResNet50, whereas MobileNet v3 Small and Large show not enough accuracy, even with an increased size (141∼391% of MobileNet v2, 13∼37% of ResNet50). MobileNet v1 also shows similar performance to MobileNet v2.

However, as shown in Table 12, MobileNet v3 shows a better performance in accuracy than MobileNet v2 in the large dataset, such as ImageNet [12]. The differences in performance between MobileNet v3 with different numbers of parameters are due to differences in training methods, in which training with large parameters in the small dataset introduces an overfitting problem.

On the other hand, the performances of WRN [20] are shown in Table 13. Even though WRN does not utilize the light-weight convolution schemes such as DSP, Linear Bottleneck and SE, it shows the best accuracy with the minimal size. Based on the aforementioned observation, it is recommended that the neural network model is selected with considering the scale of datasets.

For the performance evaluation of the KD technique, ResNet54 and MobileNet series are respectively used as teacher and student models in the CIFAR10 dataset. In addition, in order to investigate the combination with the KD and the other model optimizations (quantization and pruning), F16 and PR are used as the representative techniques.

As shown in Table 14, the KD scheme has the benefit of boosting the accuracy. In other words, although the KD itself could not minimize the size or the latency, it could increase the accuracy of lightened models for stable deployment. Moreover, if quantization and pruning are used together in the KD technique, the effect can be further enhanced. As shown in Table 8, Table 9, Table 10, Table 11 and Table 12, it is remarkable that M3L and M3s show better performance in a large-scale dataset such as ImageNet than in a small-scale dataset such as CIFAR10. This is because the M3L and M3s are designed to have the best performance in ImageNet based on NAS, i.e., it can have an inferior performance in other datasets. Therefore, when applying the KD training into those M3L and M3s models for the small-scale dataset, their performance improvement was also limited compared to the other neural networks.

### 4.3. Optimization Strategy

First, let us define the service types of AIoT and find out about the optimal DLMO strategy for each service.

The AIoT service performs existing AI services at the edge device level without going through the cloud server. According to the performance requirements, it can be classified into the following three categories:

- **Low-end AIoT Service:** Aims for low-end AIoT service such as vacant parking space detection. Their deep learning models are loaded on the small memory of the IoT device in each parking lot, but the service is not delay-sensitive. In addition, the scale of the required dataset is small, e.g., two classes with vacant and occupied classes:
- **Mid-end AIoT Service:** Aims for Mid-end AIoT Service, such as license plate recognition. The number of classes to be recognized is around 10∼20, and the difficulty of recognition is easy. In addition, the model is normally embedded on IoT devices, and real-time performance is required.
- **High-end AIoT Service:** Aims for High-end AIoT Service, such as autonomous driving. Both real-time performance and accuracy are required.

Then, based on the aforementioned remarks, the following strategies are proposed for each AIoT service:

- **Neural Network Selection:** The neural network model for DLMO needs to be selected considering the scale of the datasets.
- **Low-end AIoT Service:** It is recommended to utilize PRQ because it can minimize the model size with minimal accuracy drops.
- **Mid-end AIoT Service:** It is recommended to utilize PRN because it guarantees all performances of accuracy, latency and size reduction.
- **High-end AIoT Service:** It is better to use F16 or PRN because it guarantees all performances of accuracy, latency and size reduction.
- **KD:** For the low-to-high end AIoT services, KD can be used simultaneously with the aforementioned techniques for boosting the accuracy of the reduced model.

## 5. Conclusions

In this paper, we analyzed four methods, i.e., Light Convolution, Quantization, Pruning and Knowledge Distillation, for DLMO of edge devices and also derived application strategies according to AI services through experiments. First of all, we found that quantization was the most effective in compressing the model size, but it led to a lot of delay in data conversion. We also found that the pruning technique was excellent in all aspects of model compression, accuracy loss minimization and delay minimization. In particular, the larger the model is, the more effects of model is. Moreover, it is recommended to train the aforementioned deep learning models with knowledge distillation, because it could improve the accuracy without additional increases in latency and size. We found that it was better to select an optimized network after analyzing the data set in the field to be used, rather than selecting the lightweight network technique unconditionally according to insights from this paper. Finally, by classifying AIoT services according to three performance factors (these are accuracy, size, and delay), we derived the optimal combinations of DLMO techniques depending on situations. Moreover, transformer-based approaches [73] have recently become a dominant deep learning method instead of CNNs. However, what we have researched in this paper will be valid for a while because transformer network models are very complicated and require high hardware specifications and are thus not suitable for IoT and embedded devices.

## Figures and Tables

**Table 1:** Summary of the related work.

Reference #	Proposed
Lin et al., 2020 [17]	A framework that jointly designs the efficient neural architecture and the lightweight inference engine, enabling ImageNet-scale inference on microcontrollers (MCUNet v1).
Lin et al., 2021 [18]	A generic patch-by-patch inference scheduling, which operates only on a small spatial region of the feature map and significantly cuts down the peak memory (MCUNet v2).
Tan et al., 2019 [24]	A new scaling method that uniformly scales all dimensions of depth/width/resolution using a simple effective compound coefficient (EfficientNet).
Bello et al., 2021 [25]	Training and scaling strategies: (1) scale model depth; (2) increase image resolution depending on the training regime.
David et al., 2021 [27]	A model-architecture framework that enables hardware vendors to provide platform-specific optimizations and is open to a wide machine-learning ecosystem (TensorFlow Lite Micro).
Lai et al., 2018 [28]	An efficient kernels developed to maximize the performance and minimize the memory footprint of neural network applications on Arm Cortex-M processors targeted for intelligent IoT edge devices(CMSIS-NN)
Gural et al., 2019 [29]	Memory-optimal direct convolutions as a way to push classification accuracy as high as possible given strict hardware memory constraints at the expense of extra compute.
Sakr et al., 2021 [30]	An in-place computation strategy to reduce memory requirements of neural network inference.
Müksch et al., 2020 [31]	A comparison among several CNN variations (as like ProtoNN, Bonsai and FastGRNN) to apply 3-channel image classification using CIFAR10.

**Table 2:** KD Classification according to Knowledge Type.

Category	Meaning
Response	logit outputs of TSA
Feature	intermediate representations of TSA
Relation	relation between the feature maps

**Table 3:** KD Classification according to Distillation Type.

Category	Meaning
Offline	KD from a pre-trained teacher model
Online	Update the TSM simultaneously
Self-Distillation	Online method with same TSA

**Table 4:** KD Classification according to Teacher–Student Architecture.

Category	Meaning
Same as Teacher	Same architecture with Teacher
Reduced Teacher	Reduced architecture from Teacher
Light Network	Design with Light-Weight Conv,
	Quantization and Pruning

**Table 5:** VGGNet.

DLMO	CIFAR10			CIFAR100		
Eval	*Acc*	*Size*	*Lat*	*Acc*	*Size*	*Lat*
Setup	(%)	(KB)	(ms)	(%)	(KB)	(ms)
NQ	71.9	56	4	43.5	425	18
BLQ	71.9	19	20	43.7	112	35
FIQ	72.5	18	44	43.5	110	53
F16	72.1	30	6	43.8	214	19
QAT	72.5	18	42	42.5	106	45
PRN	68.7	16	2	39.7	123	2
PRQ	68.6	7	19	39.7	37	19

**Table 6:** ResNet50.

DLMO	CIFAR10			CIFAR100		
Eval	*Acc*	*Size*	*Lat*	*Acc*	*Size*	*Lat*
Setup	(%)	(KB)	(ms)	(%)	(KB)	(ms)
NQ	80.7	94,052	177	36.5	94,790	129
BLQ	80.3	24,161	2348	36.5	24,346	2642
FIQ	80.4	24,269	2078	37.2	24,454	1997
F16	80.6	47,072	152	39.4	47,441	162
QAT	80.5	24,281	2069	36.9	24,431	2001
PRN	81.2	27,503	155	43.2	27,857	148
PRQ	81.2	8023	2399	43.1	8059	2379

**Table 7:** ResNet101.

DLMO	CIFAR10			CIFAR100		
Eval	*Acc*	*Size*	*Lat*	*Acc*	*Size*	*Lat*
Setup	(%)	(KB)	(ms)	(%)	(KB)	(ms)
NQ	79.6	169961	336	41.3	170,698	365
BLQ	79.5	43776	4576	41.5	43,960	4646
FIQ	80.8	43990	4555	37.4	44,174	4726
F16	80.2	85072	329	41.4	85,441	352
QAT	80.1	43799	4529	37.0	44,111	4731
PRN	79.8	49704	347	38.7	49,697	337
PRQ	79.8	14812	4603	38.6	14,670	4587

**Table 8:** MobileNet v1.

DLMO	CIFAR10			CIFAR100		
Eval	*Acc*	*Size*	*Lat*	*Acc*	*Size*	*Lat*
Setup	(%)	(KB)	(ms)	(%)	(KB)	(ms)
NQ	81.8	12,840	18	42.2	13,208	34.6
BLQ	81.8	3477	346	42.2	3560	379.7
FIQ	82.4	3517	332	43.1	3612	330
F16	80.9	6435	22	45.1	6620	30.4
PRQ	81.0	1319	350	46.1	1349	350
PRN	81.0	3934	18	45.9	4063	19

**Table 9:** MobileNet v2.

DLMO	CIFAR10			CIFAR100		
Eval	*Acc*	*Size*	*Lat*	*Acc*	*Size*	*Lat*
Setup	(%)	(KB)	(ms)	(%)	(KB)	(ms)
NQ	81.6	8924	11	45.7	9386	21.7
BLQ	81.5	2663	224	45.5	2782	213.6
FIQ	81.2	2730	173	45.9	2848	161
F16	82.0	4509	10.6	41.5	4740	21.6
PRQ	80.2	1082	213	47.2	1118	221
PRN	80.5	2876	8	47.1	2997	7

**Table 10:** MobileNet v3 small.

DLMO	CIFAR10			CIFAR100		
Eval	*Acc*	*Size*	*Lat*	*Acc*	*Size*	*Lat*
Setup	(%)	(KB)	(ms)	(%)	(KB)	(ms)
NQ	68.8	12,161	10	34.8	12,624	24
BLQ	68.7	3269	73	34.9	3389	97
FIQ	69.7	3301	41	35.4	3419	43
F16	72.1	6128	10	35.2	6359	20
PRQ	69.6	1172	64	36.0	1187	69
PRN	69.7	3740	6	36.2	3862	7

**Table 11:** MobileNet v3 Large.

DLMO	CIFAR10			CIFAR100		
Eval	*Acc*	*Size*	*Lat*	*Acc*	*Size*	*Lat*
Setup	(%)	(KB)	(ms)	(%)	(KB)	(ms)
NQ	77.9	34,939	26	37.1	35,401	37
BLQ	78.0	9122	195	37.2	9239	218
FIQ	77.2	9178	149	36.7	9295	150
F16	77.9	17,525	26	38.1	17,756	28
PRQ	75.7	3062	206	39.4	3122	211
PRN	75.6	10,510	19	39.3	10,653	29

**Table 12:** MobileNet v2, v3 in ImageNet.

Network	Top-1	MAdds	Params
V3-Large1.0	75.2	219	5.4M
V2 1.0	72.0	300	3.4M
V3-Small 1.0	67.4	56	2.5M

**Table 13:** Wide Residual Networks.

DLMO	CIFAR10			CIFAR100		
Eval	*Acc*	*Size*	*Lat*	*Acc*	*Size*	*Lat*
Setup	(%)	(KB)	(ms)	(%)	(KB)	(ms)
NQ	88.8	1899	39	60.5	192	50
BLQ	88.8	537	1820	59.4	544	1860
FIQ	88.7	538	1913	59.3	544	2009
F16	86.7	982	39	60.0	994	51
QAT	88.6	540	1916	58.9	549	2002
PRN	87.6	547	42	60.2	572	37
PRQ	87.6	195	1902	60.3	200	1860

**Table 14:** Performance Evaluation of KD in the CIFAR10 Dataset.

DLMO	Original			KD		
Eval	*Acc*	*Size*	*Lat*	*Acc*	*Size*	*Lat*
Setup	(%)	(KB)	(ms)	(%)	(KB)	(ms)
M1	81.8	12,840	18	86.3	12,840	18
M2	81.6	8924	11	85.4	8924	11
M3L	77.9	34,939	26	60.5	34,939	26
M3s	68.8	12,161	10	60.5	12,161	10
M1-F16	80.9	6435	22	86.3	192	50
M2-F16	82.0	4509	10.6	85.4	192	50
M1-PR	81.0	3934	18	86.3	192	50
M2-PR	80.5	2876	8	85.4	192	50

## Data Availability

Not applicable.
